# (F, K)-Co-Doped Carbon Nitride for Enhanced Photocatalytic Hydrogen Production

**DOI:** 10.3390/nano15131021

**Published:** 2025-07-01

**Authors:** Fuhong Bi, Guiming Ba, Junbo Yu, Huilin Hu, Jinhua Ye, Defa Wang

**Affiliations:** 1 Advanced Catalytic Materials Research Center, School of Material Science and Engineering, Tianjin University, Tianjin 300072, China; 2021208241@tju.edu.cn (F.B.); bgm_123@tju.edu.cn (G.B.); 2021208151@tju.edu.cn (J.Y.); huhuilin@tju.edu.cn (H.H.); 2 State Key Laboratory of Precious Metal Functional Materials, Tianjin University, Tianjin 300072, China

**Keywords:** photocatalysis, hydrogen evolution, carbon nitride, F, K co-doping, synergistic effect

## Abstract

Visible-light-driven photocatalytic hydrogen production is one of the ideal green technologies for solar-to-chemical energy conversion. Carbon nitride (C_3_N_4_, CN) has been attracting extensive attention for its suitable band structure and stability, but the efficiency of photocatalytic hydrogen evolution is low due to insufficient visible-light absorption and rapid charge recombination. Herein, we develop a novel (F, K)-co-doped CN (FKCN) catalyst via a facile thermal polymerization approach using KOH-modified melamine and NH_4_F as the dopant precursors. The FKCN catalyst demonstrates broadened light absorption, significantly enhanced charge separation, and excellent cyclic stability. And the optimal F_(0.15)_K_(6)_CN catalyst achieves a hydrogen evolution rate of as high as 3101.5 μmol g^−1^ h^−1^ (12-fold that of pristine CN) under visible-light irradiation (λ ≥ 420 nm), which is among the best element-doped CN photocatalysts. This work highlights the effectiveness of a multi-element doping strategy in designing CN-based photocatalysts for efficient hydrogen evolution.

## 1. Introduction

In addressing the energy shortage crisis and environmental pollution, photocatalytic water splitting to generate hydrogen has emerged as one of the promising approaches for sustainable solar-to-chemical energy conversion [1,2], and the development of highly active and low-cost photocatalysts is of great significance [3]. Carbon nitride (C_3_N_4_, CN) has attracted significant attention in photocatalytic water splitting due to its unique sp^2^-hybridized conjugated structure and suitable band gap (~2.7 eV), which endows it with excellent photochemical stability and visible-light responsiveness [4,5,6]. However, the application of pristine CN in water splitting is limited by the restricted optical absorption edge (λ ≤ 460 nm), rapid recombination of photogenerated charge carriers, and smaller number of reactive surface sites [7,8,9].

To address these intrinsic drawbacks of pristine CN, various strategies have been developed, such as elemental doping [10], heterojunction construction [11], surface functional group modification [12], nanostructural design [13], etc. Among these approaches, elemental doping was used as an effective method to enhance the photocatalytic activity of CN, as it could tune the electronic structure, increase the active site density, and facilitate charge carrier separation/transfer [14,15]. Specifically, potassium (K) doping could be inserted into the CN interlayers to facilitate interlayer electron transfer and generate cyano groups to provide additional active sites [16]. For instance, Sun et al. synthesized K-doped CN ultrathin nanosheets via a KOH-assisted hydrothermal melamine method, leading to an improved hydrogen evolution performance, which was attributed to an increased specific surface area, upward-shifted conduction band edge for enhanced photoreduction ability, and prolonged charge carrier lifetime [17]. Wang et al. reported K-doped g-C_3_N_4_ using potassium bromide (KBr) as the dopant source through thermal polymerization, with the optimized K-CN-10 catalyst demonstrating a hydrogen evolution rate of 1337 μmol g^−1^ h^−1^ [18]. The enhancement was ascribed to a reduced band gap, improved light-harvesting capacity, and enhanced electron transport. Chang et al. introduced K atoms and cyano functional groups into CN via potassium thioacetate-mediated thermal polymerization, achieving a hydrogen evolution rate of 1319 μmol g^−1^ h^−1^ for the optimized K(0.05)-CN catalyst [19]. The improved performance was attributed to an increased electron density in heptazine ring delocalized π bonds from K doping and lone-pair electron delocalization by cyano groups.

To further improve the photocatalytic performance of K-doped CN, other elements were often co-doped with K to jointly modify the electronic structure of CN. For example, Guo et al. fabricated K/I-co-doped mesoporous CN using dicyandiamide and KI as precursors with SBA-15 as a hard template via thermal polymerization [20]. The optimized MP-CN-KI catalyst achieved a hydrogen evolution rate of 1611 μmol g^−1^ h^−1^, which was attributed to a prolonged carrier lifetime, improved electrical conductivity, and enhanced photocurrent density. Bi et al. synthesized S/K-co-doped CN through a condensation reaction of thiourea and dithiooxamide, with the optimized CN-0.20%Dx-25 catalyst demonstrating a hydrogen evolution rate of 1962 μmol g^−1^ h^−1^ [21]. This improvement was ascribed to K atoms creating charge transport channels between CN layers and synergistic effects of S/K co-doping that introduced additional electrons into the band gap, forming a metalloid-like band structure.

It is noteworthy that fluorine (F) has a smaller atomic radius and higher electronegativity compared to other dopant atoms, making it easier to be doped into CN and modifying its electronic structure. Herein, we report a (F, K)-co-doped CN (FKCN) photocatalyst synthesized through a novel thermal polymerization approach for enhanced photocatalytic H_2_ evolution. Synergistic integration of KOH-modified melamine with NH_4_F in a reaction system enables precise control over structural defects and electronic properties of FKCN. This synthetic route not only overcomes the limitations of conventional doping methods but also creates a new paradigm for designing high-efficiency photocatalytic materials with tailored energy band structures. This work demonstrates an innovative way via the integration of alkali metal and halogen co-doping of developing efficient photocatalysts with an enhanced light-harvesting capability and charge carrier separation efficiency.

## 2. Materials and Methods

### 2.1. Materials and Synthesis

Melamine (99%, Shanghai Aladdin Biochemical Technology Co., Ltd., Shanghai, China) was employed for the synthesis of the CN skeleton. Ammonium fluoride (98%, Sinopharm Chemical Reagent Co., Ltd., Shanghai, China) and potassium hydroxide (82%, Sinopharm Chemical Reagent Co., Ltd.) served as doping sources. Methanol (99.5%, Tianjin Anlong Bohua Medicinal Chemistry Co., Ltd., Tianjin, China) was utilized as the sacrificial agent. All reagents in the experiment were analytical-grade and used without further purification

Pristine CN was synthesized through thermal polymerization of melamine precursors. Specifically, 4.0 g of melamine was placed in a ceramic crucible and subjected to thermal treatment under an argon atmosphere (50 mL min^−1^). The temperature was heated to 823 K at a ramping rate of 5 K min^−1^ and maintained at this temperature for 4 h. The resulting pale-yellow product was obtained after natural cooling to an ambient temperature.

F-doped CN (F_(x)_CN) was fabricated via a solution impregnation–calcination strategy. In a typical procedure, 0.5 g of melamine and different amounts (x = 0.1, 0.125, 0.15, 0.175, 0.2 g) of ammonium fluoride (NH_4_F) were co-dissolved in 10 mL of deionized water. The homogeneous solution was continuously stirred at 353 K until complete solvent evaporation. The dried mixture was subsequently calcined under identical conditions to those for pristine CN synthesis. The obtained samples were named F_(x)_CN (x = 0.1, 0.125, 0.15, 0.175, 0.2).

**Figure 1:**
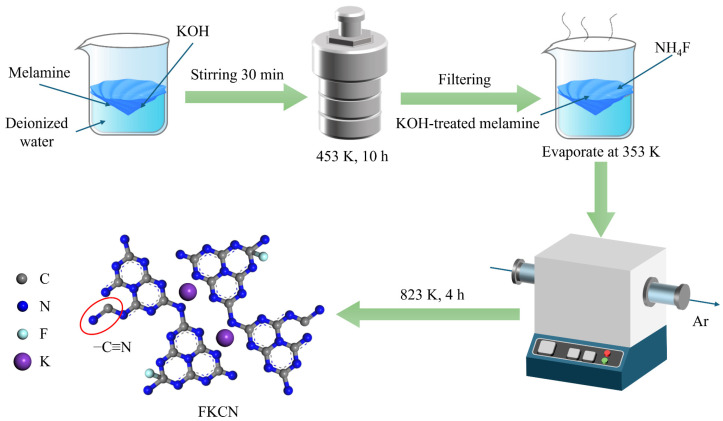
Schematic illustration for the preparation of (F, K)-co-doped CN photocatalyst.

K-doped CN (K_(y)_CN) was prepared through a hydrothermal approach, where 8 g of melamine and different amounts (y = 2, 4, 6, 8 g) of potassium hydroxide (KOH) were dissolved in 70 mL deionized water and hydrothermally treated at 453 K for 12 h in a Teflon-lined autoclave. The resulting slurry was vacuum-filtered and dried at 333 K for 12 h. The obtained precursor was then calcined using the same protocol as for pristine CN. The obtained samples were named K_(y)_CN (y = 2, 4, 6, 8).

(F, K)-co-doped CN (F_(x)_K_(6)_CN) was prepared through a two-step modification approach, as illustrated in Figure 1. First, 8 g of melamine and 6 g of KOH were dispersed in 70 mL of deionized water and hydrothermally treated at 453 K for 12 h in a Teflon-lined autoclave. The resulting slurry was filtered and vacuum dried at 333 K for 12 h, obtaining K_(6)_CN. Then, 0.5 g of the pre-synthesized K_(6)_CN and different amounts (x = 0.1, 0.125, 0.15, 0.175, 0.2 g) of NH_4_F were added to 10 mL of aqueous solution under stirring and evaporated to dryness. Finally, the obtained precursor was then calcined using the same protocol as for pristine CN. The obtained samples were fully ground and denoted as F_(x)_K_(6)_CN (x = 0.1, 0.125, 0.15, 0.175, 0.2).

### 2.2. Characterization

The crystal structure was characterized by X-ray diffraction (XRD) on a D8 Advance diffractometer (Bruker Corporation, Karlsruhe, Germany) with monochromatic Cu Kα radiation (λ = 0.15418 nm), operating at a scanning range of 5–60°, a sampling interval of 0.01°, and a scanning speed of 10° min^−1^. Fourier transform infrared (FTIR) spectra were recorded on a Nicolet-6700 spectrometer (Thermo Fisher Scientific, Waltham, MA, USA) using the KBr pellet method for chemical bonding analysis, with a scanning range of 400–4000 cm^−1^, 32 scans, and a resolution of 8 cm^−1^. X-ray photoelectron spectroscopy (XPS) analysis was performed using an ESCALAB-250Xi spectrometer (Thermo Fisher Scientific, Waltham, MA, USA) to determine the elemental composition chemical state. The microstructure was observed on a field emission scanning electron microscope (FE-SEM, Hitachi S-4800, Tokyo, Japan) and a field emission transmission electron microscope (FE-TEM, Thermo Fisher Scientific, Waltham, MA, USA). The specific surface area was quantitatively determined by nitrogen adsorption–desorption isotherms at 77 K using a Quantachrome Autosorb-iQ instrument (Thermo Fisher Scientific, Waltham, MA, USA), after the sample was degassed at 357 K for 6 h. The optical absorption property was investigated on a UV-3600 spectrophotometer (Shimadzu Corporation, Kyoto, Japan). Charge carrier dynamics were probed through both steady-state photoluminescence (PL) and time-resolved PL (TRPL) using a HORIBA Fluorolog-3 spectrofluorometer (Horiba Scientific, Irvine, CA, USA) at ambient temperature, with an excitation wavelength of 391 nm. Electron paramagnetic resonance (EPR) measurements were conducted at room temperature on a JES-FA200 spectrometer (Japan Electron Optics Laboratory Co., Ltd., Tokyo, Japan).

### 2.3. Photoelectrochemical Measurements

Photoelectrochemical (PEC) properties were measured using an electrochemical workstation (CHI660E, Shanghai Chenhua, Shanghai, China) with a standard three-electrode system. The as-prepared sample served as the working electrode (effective illumination area: ~1 cm^2^), while a platinum plate counter electrode and an Ag/AgCl reference electrode were immersed in a 0.5 M Na_2_SO_4_ aqueous electrolyte. A 300 W xenon lamp was employed as light irradiation source. Mott-Schottky analysis was conducted under a dark condition at three characteristic frequencies (1000, 900, and 800 kHz) with an AC amplitude of 5 mV versus the open circuit potential, and the capacitance–voltage relationships were recorded. Electrochemical impedance spectroscopy (EIS) was carried out at the open circuit potential with a 5 mV AC perturbation over a frequency range of 100 kHz to 1 Hz. Photocurrent response curves (i–t) were acquired under intermittent illumination with periodic light “on/off” pulses (20 s) at an applied bias of 0.6 V vs. RHE.

### 2.4. Hydrogen Evolution Reaction (HER) Measurements

Photocatalytic hydrogen evolution from water was carried out in a side irradiation Pyrex glass reaction cell of 330 mL connected to a closed circulation system with an inline evacuation system. In a standard procedure, 20 mg catalyst was uniformly dispersed by a magnetic stirrer in 270 mL aqueous solution containing methanol (10 vol%) as the sacrificial agent and H_2_PtCl_6_·6H_2_O (1 wt% Pt loading) as the cocatalyst precursor. Before reaction, the closed gas circulation system and the reaction cell were well evacuated and then introduced into ~2.5 kPa of argon gas. Prior to hydrogen collection, the suspension underwent 30 min of 300 W Xe lamp irradiation (λ ≥ 420 nm) to photodeposit platinum (Pt) nanoparticles as a cocatalyst onto the photocatalyst surface. After the closed gas circulation system and the reaction cell were evacuated and introduced into ~2.5 kPa of argon gas again, the photocatalytic hydrogen evolution reaction was initiated under visible-light irradiation from a 300 W Xe lamp (CEL-HXF300, CEAU-light Co., Ltd., Beijing, China) with a 420 nm long-pass optical filter. Evolved hydrogen was measured on an online gas chromatograph (GC-2014C, Shimadzu Corporation, Kyoto, Japan) equipped with a thermal conductivity detector (TCD) using argon carrier gas.

The apparent quantum yield (*AQY*) of F_0.15_K_6_CN was calculated using the following equation:

$$AQY=\frac{N_{e}}{N_{p}}\times 100\%=\frac{\left(2\times r_{H}\times N_{A})\times (h\times c\right)}{I\times A\times \lambda }\times 100\%$$

where *N_e_* is total number of electrons transferred in the reaction, *N_p_* is the total number of incident photons, *r_H_* is the hydrogen evolution rate (mol/s), *N_A_* is Avogadro’s constant (6.02 × 1023 mol^−1^), *h* is Planck’s constant (6.62 × 10^−34^ J·s), *c* is the speed of light (3 × 10^8^ m s^−1^), *A* is the illumination area (m^2^), and *λ* is the incident light wavelength (m).

## 3. Results and Discussion

### 3.1. Structure, Morphology, and Optical Properties of Catalysts

**Figure 2:**
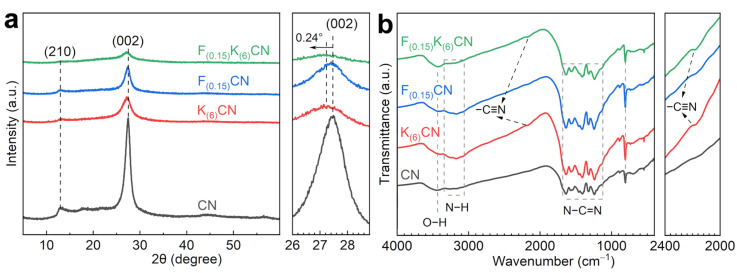
(**a**) XRD patterns and (**b**) FTIR spectra of CN, K_(6)_CN, F_(0.15)_CN, and F_(0.15)_K_(6)_CN.

The crystal structures of catalysts were analyzed by powder X-ray diffraction (XRD) (Figure 2a and Figure S1). The CN exhibited two typical diffraction peaks at 12.9° and 27.4°, corresponding to the (210) in-planar repeated heptazine units and the (002) interlayer packing structure, respectively [22]. The diffraction peak intensities of K_(6)_CN and F_(0.15)_CN were weaker than that of CN and decreased with the increase in doping amount, indicating that the introduction of K and F atoms might alter the growth of the CN crystal planes [23]. Moreover, F_(0.15)_K_(6)_CN showed weaker diffraction peaks’ intensity than K_(6)_CN and F_(0.15)_CN, signifying a more significant structural transformation after (K, F) co-doping. More importantly, the (002) diffraction peak of K_(6)_CN and F_(0.15)_K_(6)_CN shifted to lower diffraction angles than CN, indicating that the K^+^ intercalation in the CN interlayer increased the stacking distance between the nanosheets, which was favorable for the rapid interlayer transfer of the photogenerated charge carrier [24]. The functional groups of the obtained samples were examined using Fourier transform infrared (FTIR) spectroscopy (Figure 2b and Figure S2). All samples exhibited similar characteristic peaks at 805, 1127–1684, 3063–3343, and 3435 cm^−1^, which were ascribed to the bending vibration of the heptazine unit and stretching vibrations of N−C=N, N−H, and O−H, respectively, indicating that K and/or F element doping did not change the basic framework of CN [25]. From the zoomed display in Figure 2b, a new characteristic peak at 2175 cm^−1^ assigned to the asymmetric stretching vibration of cyano groups (−C≡N) was observed in K_(6)_CN and F_(0.15)_K_(6)_CN. The peak intensity increased with the increase in K doping amount, indicating that K doping led to the opening of the heptazine ring [26]. The strong electron-withdrawing property of −C≡N was conducive to charge separation [27]. In addition, no characteristic peaks related to K or F were detected in K_(6)_CN, F_(0.15)_CN, and F_(0.15)_K_(6)_CN, due to the too low doping amounts of these elements.

**Figure 3:**
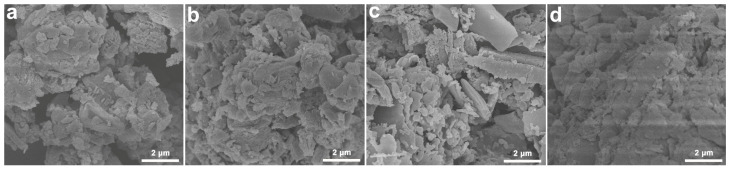
SEM images: (**a**) CN, (**b**) K_(6)_CN, (**c**) F_(0.15)_CN, and (**d**) F_(0.15)_K_(6)_CN.

The SEM image of pristine CN showed a roughly aggregated nanostructure (Figure 3a). After the introduction of K atoms, K_(6)_CN maintained the same morphology as CN (Figure 3b). However, F_(0.15)_CN exhibited a large number of nanosheet structures with a relatively smooth surface (Figure 3c). Interestingly, after (K, F) co-doping, F_(0.15)_K_(6)_CN was composed of a roughly aggregated nanostructure and smooth-surfaced nanosheets (Figure 3d). TEM images also showed a similar layered stacking structure for all samples (Figure S3). Specifically, K_(6)_CN featured a relatively loose and rough surface, F_(0.15)_CN had a smoother surface, and F_(0.15)_K_(6)_CN presented a morphology in between K_(6)_CN and F_(0.15)_CN. The specific surface area and pore size of CN, K_(6)_CN, F_(0.15)_CN, and F_(0.15)_K_(6)_CN were measured by N_2_ adsorption-desorption isotherms (Figure S4a). All samples exhibited type IV isotherms with H3 hysteresis loops, demonstrating that these nanosheets were mesoporous materials. The specific surface areas of F_(0.15)_K_(6)_CN (23.2 m^2^ g^−1^), K_(6)_CN (22.0 m^2^ g^−1^), and F_(0.15)_CN (22.9 m^2^ g^−1^) were similar but higher than that of CN (17.9 m^2^ g^−1^), consistent with the SEM observations. Furthermore, the number of pore sizes within the range of 3–10 nm in F_(0.15)_K_(6)_CN was also similar to those in K_(6)_CN and F_(0.15)_CN but higher than that in CN (Figure S4b), indicating that K and/or F doping resulted in the formation of more mesopores. Importantly, the increased BET surface area and mesopores might provide more active sites for improved photocatalytic activity [28].

**Figure 4:**
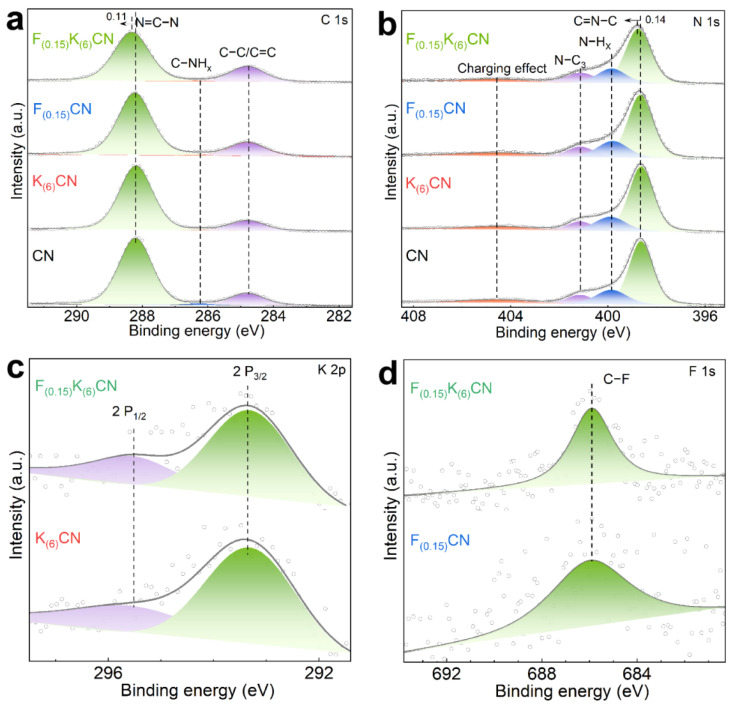
(**a**) C 1s and (**b**) N 1s high-resolution XPS spectra of CN, K_(6)_CN, F_(0.15)_CN, and F_(0.15)_K_(6)_CN. (**c**) K 2p high-resolution XPS spectra of K_(6)_CN and F_(0.15)_K_(6)_CN. (**d**) F 1s high-resolution XPS spectra of F_(0.15)_CN and F_(0.15)_K_(6)_CN.

The surface compositions and chemical states of CN, K_(6)_CN, F_(0.15)_CN, and F_(0.15)_K_(6)_CN were investigated by X-ray photoelectron spectroscopy (XPS). As shown in Figure 4a, the C 1s high-resolution XPS spectrum of CN could be deconvoluted into three peaks at binding energies of 284.8, 286.2, and 288.2 eV, corresponding to graphitic carbon (C−C/C=C), C−NH_x_ on the edges of heptazine units, and sp^2^-hybridized carbon (N=C−N), respectively [29]. In the N 1s high-resolution XPS spectrum of CN (Figure 4b), four distinct peaks appeared at binding energies of 398.7, 399.9, 401.2, and 404.6 eV, which were attributed to sp^2^-hybridized nitrogen (C=N−C), the amino functional group (N−H_x_), tertiary nitrogen (C−N_3_), and the charging effect, respectively [30,31]. The C 1s and N 1s XPS spectra of both K_(6)_CN and F_(0.15)_CN were similar to that of CN. However, the peak positions of N=C−N in the C 1s spectrum and C=N−C in the N 1s spectrum of F_(0.15)_K_(6)_CN shifted to a higher binding energy than those of CN. It indicated that (K, F) co-doping was more likely to modify the electronic structure of CN compared to sole doping with K or F, resulting in the generation of more dissociated electrons. For the O 1s high-resolution XPS spectra (Figure S5), all samples exhibited a similar characteristic peak of adsorbed water at 532.4 eV, indicating that the K or F doping did not introduce additional oxygen elements [32]. The K 2p high-resolution XPS spectra of K_(6)_CN and F_(0.15)_K_(6)_CN exhibited two prominent peaks at 293.4 and 295.5 eV (Figure 4c), corresponding to K 2p_3/2_ and K 2p_1/2_, respectively [33]. As shown in Figure 4d, a clear peak at 685.9 eV in the F 1s high-resolution XPS spectra was observed for F_(0.15)_CN and F_(0.15)_K_(6)_CN, which was assigned to the coordination of the C−F bond [34]. We need to note that due to the intercalation of K^+^ into the interlayers of CN, the interlayer spacing was expanded, allowing a large amount of F^−^ with a smaller ionic radius to be incorporated into CN. As a result, F_(0.15)_K_(6)_CN exhibited a more intense peak than F_(0.15)_CN, the latter of which showed a decrease in the signal-to-noise ratio, an increase in baseline noise of the peak, and a “diffuse” peak shape possibly caused by statistical fluctuations. Similar phenomena have been observed in previous reports [35,36]. The above results indicated that K atoms were effectively doped into the CN interlayer, while F atoms were incorporated into the CN framework through the formation of C−F bonds.

**Figure 5:**
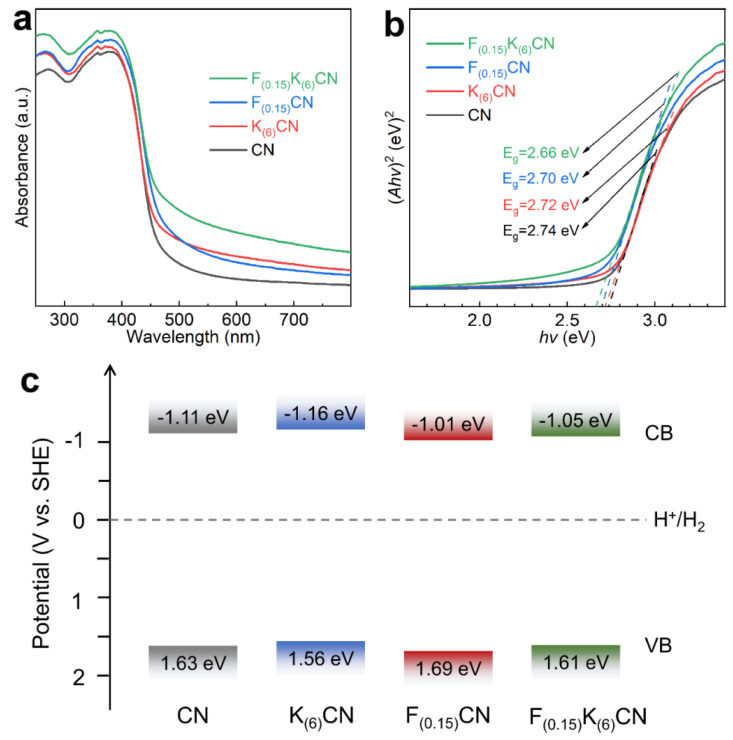
(**a**) UV-vis diffuse reflectance spectra, (**b**) corresponding Tauc plot, and (**c**) band structure alignments of CN, K_(6)_CN, F_(0.15)_CN, and F_(0.15)_K_(6)_CN.

The effects of K and F co-doping on the optical properties of CN were analyzed using UV–vis diffuse reflectance spectroscopy. As shown in Figure 5a, K_(6)_CN, F_(0.15)_CN, and F_(0.15)_K_(6)_CN exhibited an increased light capture capability compared to CN over the full wavelength range of 250–800 nm. More importantly, the light absorption capability of F_(0.15)_K_(6)_CN was significantly higher than that of K_(6)_CN or F_(0.15)_CN, revealing that (K, F) co-doping could more easily optimize the electronic structure of CN, which was consistent with the XPS results. Furthermore, the optical band gaps (E_g_) of CN, K_(6)_CN, F_(0.15)_CN, and F_(0.15)_K_(6)_CN were determined by the Tauc plot from the Kubelka–Munk function conversion to be 2.74, 2.72, 2.70, and 2.66 eV, respectively (Figure 5b). In order to clarify the band structure of samples, the flat band potentials were calculated according to the Mott−Schottky plots (Figure S6). Notably, the obtained positive slopes confirmed that all samples were typical of an n-type semiconductor. Therefore, the conduction band potential (E_CB_) values of CN, K_(6)_CN, F_(0.15)_CN, and F_(0.15)_K_(6)_CN were approximately equal to their flat band potentials of −1.11, −1.16, −1.01, and −1.05 eV, respectively [37]. The corresponding valence band potential (E_VB_) values of CN, K_(6)_CN, F_(0.15)_CN, and F_(0.15)_K_(6)_CN were calculated using the equation E_VB_ = E_g_ + E_CB_ to be 1.63, 1.56, 1.69, and 1.61 eV, respectively [38]. On the basis of the above results, the band structures of CN, K_(6)_CN, F_(0.15)_CN, and F_(0.15)_K_(6)_CN are illustrated in Figure 5c, which satisfied the requirements for photocatalytic water splitting to produce H_2_.

### 3.2. Photocatalytic H_2_ Evolution Performance

**Figure 6:**
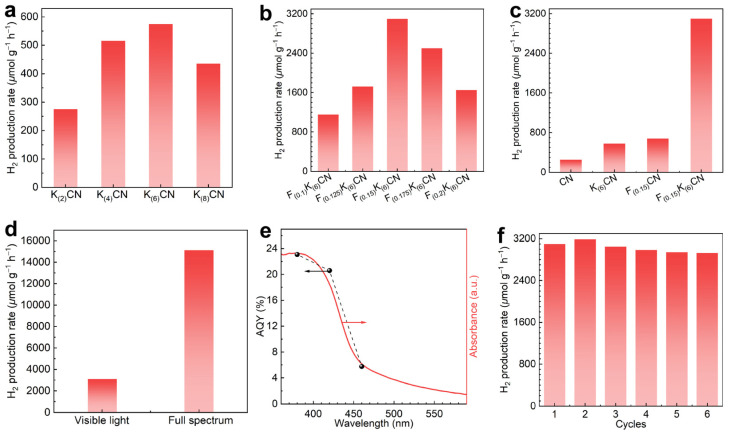
The H_2_ production rates over (**a**) K_(y)_CN (y = 2, 4, 6, 8), (**b**) F_(x)_K_(6)_CN (x = 0.1, 0.125, 0.15, 0.175, 0.2), and (**c**) CN, K_(6)_CN, F_(0.15)_CN, and F_(0.15)_K_(6)_CN under visible-light irradiation. (**d**) Comparison of H_2_ production rates over F_(0.15)_K_(6)_CN under visible-light and full-spectrum-light irradiation. (**e**) The wavelength-dependent AQY of F_(0.15)_K_(6)_CN for photocatalytic H_2_ production. (**f**) The cycling experiment of F_(0.15)_K_(6)_CN.

Photocatalytic hydrogen production was carried out using methanol as a hole scavenger under visible-light irradiation (λ ≥ 420 nm). As shown in Figure 6a, the photocatalytic activities of K_(y)_CN (y = 2, 4, 6, 8) initially increased and then decreased when increasing the K doping concentration, and K_(6)_CN exhibited the highest H_2_ production rate. For the (K, F)-co-doped samples F_(x)_K_(6)_CN (x = 0.1, 0.125, 0.15, 0.175, 0.2), the optimal F_(0.15)_K_(6)_CN exhibited the highest H_2_ production rate of 3101.5 μmol g^−1^ h^−1^ (Figure 6b). In addition, Figure 6c shows that the H_2_ production rates of K_(6)_CN (575 μmol g^−1^ h^−1^) and F_(0.15)_CN (680 μmol g^−1^ h^−1^) were approximately 2.3 and 2.7 times that of CN (255 μmol g^−1^ h^−1^), respectively. Notably, the H_2_ production rate of F_(0.15)_K_(6)_CN was about 12.2 times that of CN, indicating a significant synergistic effect of (K, F) co-doping in enhancing photocatalytic activity. Moreover, the H_2_ production rate of F_(0.15)_K_(6)_CN could be increased from 3101.5 to 15,124 μmol g^−1^ h^−1^ under full-spectrum-light irradiation (Figure 6d). It is worth noting that the photocatalytic H_2_ production performance of F_(0.15)_K_(6)_CN surpassed those of most recently reported element-doped CN photocatalysts (Table S1). We also conducted additional photocatalytic activity tests using Cu, Fe, and Ni as the cocatalysts, which showed apparently improved activities but not as good as that when using Pt as the cocatalyst (Figure S7).

The apparent quantum yields (AQYs) of F_(0.15)_K_(6)_CN for photocatalytic H_2_ production were measured under various forms of monochromatic light irradiation. As shown in Figure 6e, the AQY values at wavelengths of 380, 420, and 460 nm were determined to be 23.1, 20.6, and 5.8%, respectively, consistent with the absorption spectrum. This result strongly suggested the nature of photocatalytic H_2_ production over F_(0.15)_K_(6)_CN. The stability of the photocatalytic H_2_ production of F_(0.15)_K_(6)_CN was evaluated through six cycling tests. As shown in Figure 6f, the H_2_ production rate of F_(0.15)_K_(6)_CN exhibited no significant decline, indicating its excellent stability in photocatalytic activity. In addition, the robust structural stability of F_(0.15)_K_(6)_CN was confirmed through the nearly unchanged XRD patterns (Figure S8a) and FTIR spectra (Figure S8b) before and after the cycling tests.

### 3.3. Mechanistic Investigation of the Enhanced Photocatalytic H_2_ Production over F_(0.15)_K_(6)_CN

In order to obtain evidence of the electronic structural changes caused by (K, F) co-doping, the electron paramagnetic resonance (EPR) spectra were measured to detect the spin state of unpaired electrons (Figure S9). All samples displayed a single Lorentzian line with an essentially same g value of 2.004, corresponding to unpaired electrons from the sp^2^-carbon atoms of π-conjugated aromatic rings [39]. Clearly, the EPR signal intensity of F_(0.15)_K_(6)_CN was significantly higher than those of CN, K_(6)_CN, and F_(0.15)_CN, indicating that F_(0.15)_K_(6)_CN had a higher concentration of unpaired electrons. This was probably because the insertion of K atoms and the formation of C−F bonds altered the symmetrical electronic structure of the catalyst, which was beneficial for the separation and migration of photogenerated charge carriers.

**Figure 7:**
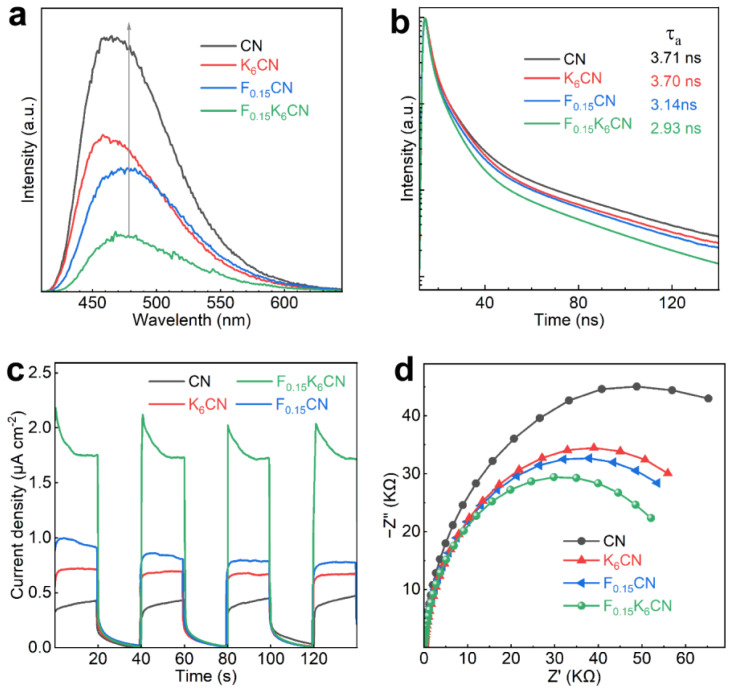
(**a**) Steady-state PL spectra, (**b**) time-resolved PL spectra, (**c**) photocurrent response curves, and (**d**) EIS Nyquist plots of CN, K_(6)_CN, F_(0.15)_CN, and F_(0.15)_K_(6)_CN.

To investigate the separation and transfer behaviors of charge carriers in photocatalysts, steady-state and time-resolved PL spectra were measured. As shown in Figure 7a, the PL emission peak at approximately 465 nm for CN originated from the radiative recombination during the decay of excited electrons in heptazine rings [40]. The PL emission peak positions of F_(0.15)_CN and F_(0.15)_K_(6)_CN were red shifted relative to that of CN, in accordance with the narrowed band gap. In addition, the PL intensity of K_(6)_CN and F_(0.15)_CN was much lower than that of CN, indicating that K or F doping could act as a trapping site to suppress the recombination of photogenerated electron-hole pairs [41]. With (K, F) co-doping, the PL intensity of F_(0.15)_K_(6)_CN was further reduced, indicating that the introduction of K and F dual sites significantly increased the separation efficiency of a photogenerated charge carrier. Meanwhile, fitting the time-resolved PL spectra revealed that F_(0.15)_K_(6)_CN exhibited a shorter average radiative lifetime (τ_a_, 2.93 ns) than CN (3.71 ns), K_(6)_CN (3.70 ns), and F_(0.15)_CN (3.14 ns), indicating enhanced photoexciton dissociation and nonradiative energy transfer efficiency due to (K, F) co-doping (Figure 7b).

The separation and transfer ability of photogenerated charge carriers was further characterized by photoelectrochemical tests. Figure 7c shows the photocurrent response curves of CN, K_(6)_CN, F_(0.15)_CN, and F_(0.15)_K_(6)_CN under on/off visible-light illumination (λ ≥ 420 nm). Clearly, F_0.15_K_6_CN exhibited the highest photocurrent density (1.72 μA cm^−2^) compared to CN (0.43 μA cm^−2^), K_(6)_CN (0.67 μA cm^−2^), and F_(0.15)_CN (0.79 μA cm^−2^), indicating that F_(0.15)_K_(6)_CN had an efficient photogenerated electron-hole pair separation ability. Moreover, the electrochemical impedance spectroscopy (EIS) spectra of the samples were measured. As shown in Figure 7d, the EIS Nyquist plot arc radius of F_(0.15)_K_(6)_CN was smaller than those of CN, K_(6)_CN, and F_(0.15)_CN, suggesting that (K, F) co-doping reduced the charge transfer resistance at the photocatalyst interface, which benefited the migration of charge carriers [42]. The above results proved that (K, F) co-doping effectively accelerated the charge separation efficiency and transport rate, thereby enhancing the photocatalytic H_2_ production activity of F_(0.15)_K_(6)_CN.

## 4. Conclusions

In summary, we successfully developed a (F, K)-co-doped carbon nitride (FKCN) catalyst via thermal polymerization of KOH-assisted hydrothermally modified melamine and NH_4_F. Characterizations revealed that K doping intercalated into CN interlayers, facilitating rapid charge carrier transport and generating cyano groups as additional active sites, while F doping formed C−F bonds, modulating the electronic structure to significantly enhance photogenerated charge separation. These synergistic effects from (F, K) doping resulted in an optimized band structure and expanded visible-light absorption. The optimal F_(0.15)_K_(6)_CN photocatalyst achieved a high hydrogen evolution rate of 3101.5 μmol g^−1^ h^−1^, which surpassed most of reported element-doped CN photocatalysts. This work demonstrates that multi-element doping is a viable strategy to design high-performance CN-based photocatalysts for sustainable hydrogen production.

## Data Availability

Data will be made available on request.

## Supplementary Materials

The following supporting information can be downloaded at: https://www.mdpi.com/article/10.3390/nano15131021/s1, Figure S1 XRD patterns of (a) CN and K_(y)_CN (y = 2, 4, 6, 8); (b) CN and F_(x)_K_(6)_CN (x = 0.1, 0.125, 0.15, 0.175, 0.2); Figure S2 FTIR spectra of (a) CN and K_(y)_CN (y = 2, 4, 6, 8); (b) CN and F_(x)_K_(6)_CN (x = 0.1, 0.125, 0.15, 0.175, 0.2); Figure S3 TEM images of (a) CN, (b) K_(6)_CN, (c) F_(0.15)_CN, and (d) F_(0.15)_K_(6)_CN; Figure S4 (a) N_2_ sorption isotherms and (b) pore size (D) distribution curves of CN, K_(6)_CN, F_(0.15)_CN, and F_(0.15)_K_(6)_CN; Figure S5 High-resolution O 1s XPS spectra of CN, K_(6)_CN, F_(0.15)_CN, and F_(0.15)_K_(6)_CN; Figure S6 Mott–Schottky plots of (a) CN, (b) K_(6)_CN, (c) F_(0.15)_CN, and (d) F_(0.15)_K_(6)_CN; Figure S7 Photocatalytic hydrogen production activities of F_(0.15)_K_(6)_CN without cocatalyst loading, and loaded with 1 wt% of Cu, Fe, and Ni; Figure S8 (a) XRD patterns and (b) FTIR spectra of F_(0.15)_K_(6)_CN before and after the cyclic experiments; Figure S9 EPR spectra of CN, K_(6)_CN, F_(0.15)_CN, and F_(0.15)_K_(6)_CN; Table S1 Photocatalytic H_2_ production performance comparison between F_(0.15)_K_(6)_CN and other reported element-doped CN photocatalysts. Refs. [17,18,19,20,21,43,44,45,46,47,48,49,50,51] are cited in the Supplementary Materials.
