# Supplementary material for: (F, K)-Co-Doped Carbon Nitride for Enhanced Photocatalytic Hydrogen Production
Source: Nanomaterials (Basel). 2025 Jul 1;15(13):1021. doi: 10.3390/nano15131021 (PMC12250847; doi:10.3390/nano15131021)
Supplement: Supplementary file 1 [file nanomaterials-15-01021-s001.zip › nanomaterials-3703013-supplementary.pdf]

Supplementary Materials

(F, K) Co-doped Carbon Nitride for Enhanced Photocatalytic Hydrogen Production

Fuhong Bi [1], Guiming Ba [1], Junbo Yu [1], Huilin Hu [1], Jinhua Ye [1,2,*] and Defa Wang [1,2,*]

1 Advanced Catalytic Materials Research Center, School of Material Science and Engineering, Tianjin University, Tianjin 300072, China
2 State Key Laboratory of Precious Metal Functional Materials, Tianjin University, Tianjin 300072, China
* Correspondence: jinhua.ye@nims.go.jp (J.Y.); defawang@tju.edu.cn (D.W.)

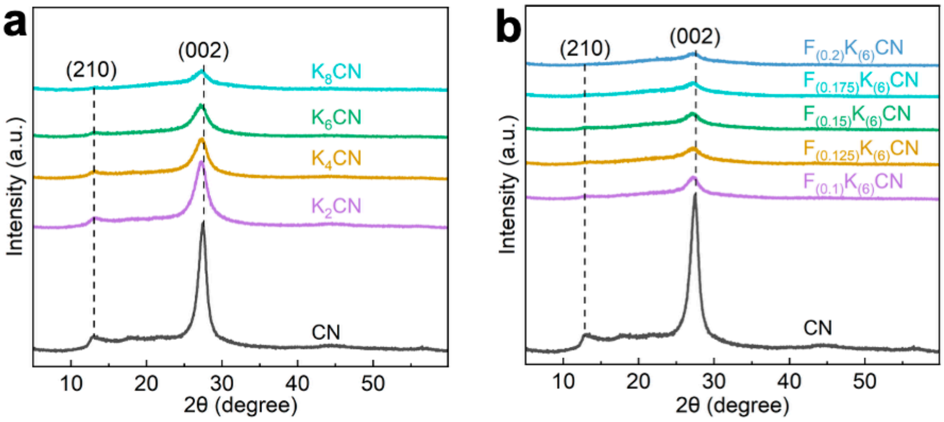

Figure S1 XRD patterns of (a) CN and $K_{(y)}CN$ (y = 2, 4, 6, 8); (b) CN and $F_{(x)}K_{(6)}CN$ (x = 0.1, 0.125, 0.15, 0.175, 0.2).

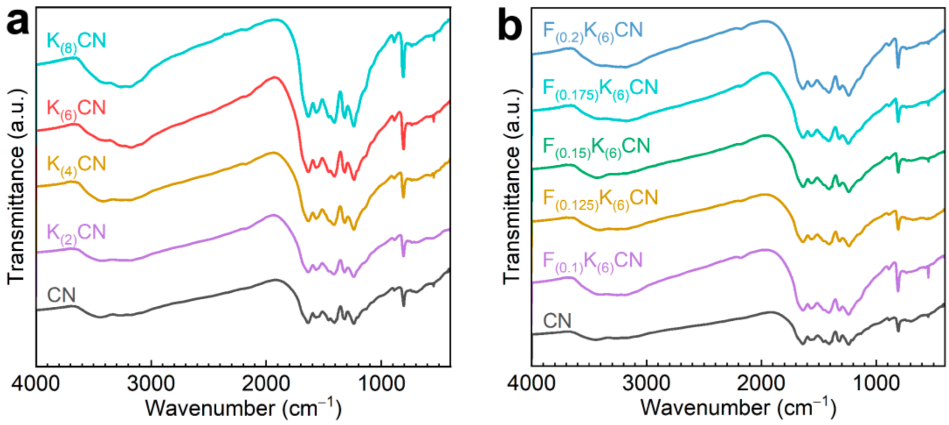

Figure S2 FTIR spectra of (a) CN and $K_{(y)}CN$ (y = 2, 4, 6, 8); (b) CN and $F_{(x)}K_{(6)}CN$ (x = 0.1, 0.125, 0.15, 0.175, 0.2).

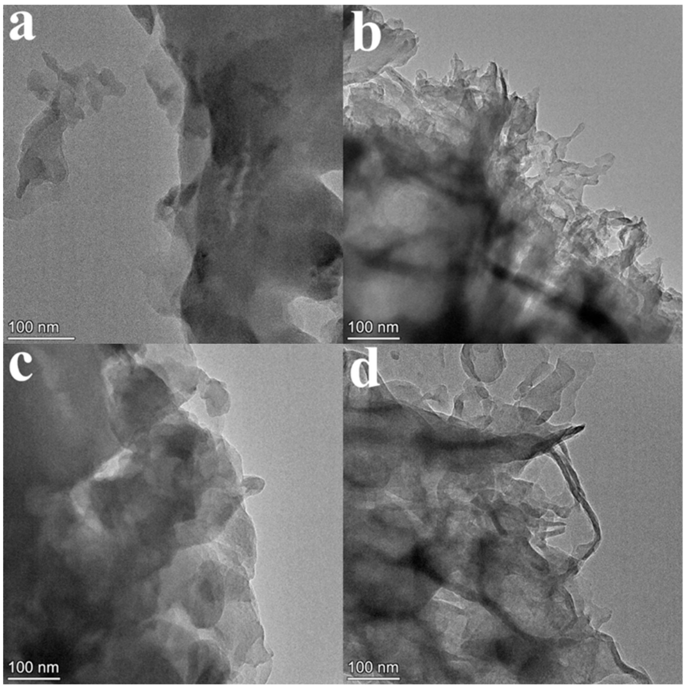

Figure S3 TEM images of (a) CN, (b) $K_{(6)}CN$, (c) $F_{(0.15)}CN$, and (d) $F_{(0.15)}K_{(6)}CN$.

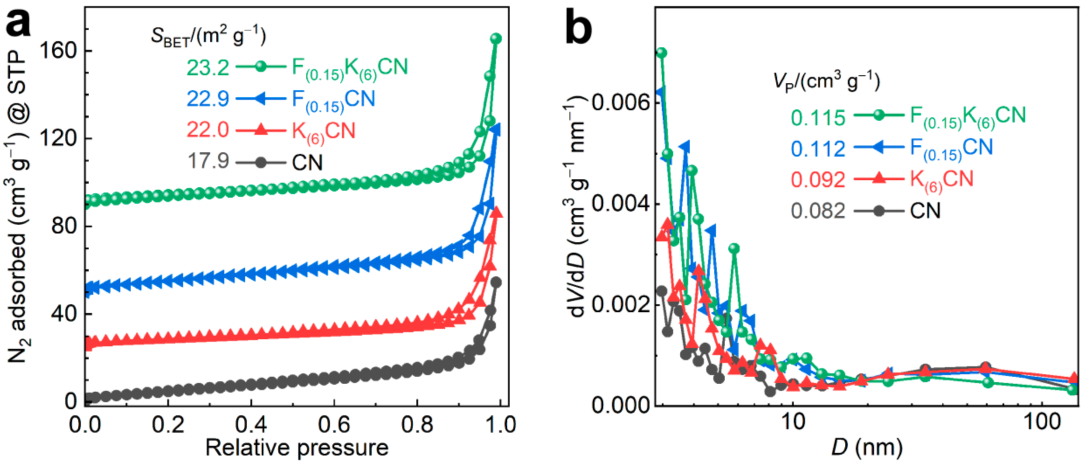

Figure S4 (a) N_2 adsorption–desorption isotherms and (b) pore size (D) distribution curves obtained from the adsorption-desorption isotherms using the Barrett-Joyner-Halenda (BJH) method for CN, $K_{(6)}$CN, $F_{(0.15)}$CN, and $F_{(0.15)}K_{(6)}$CN.

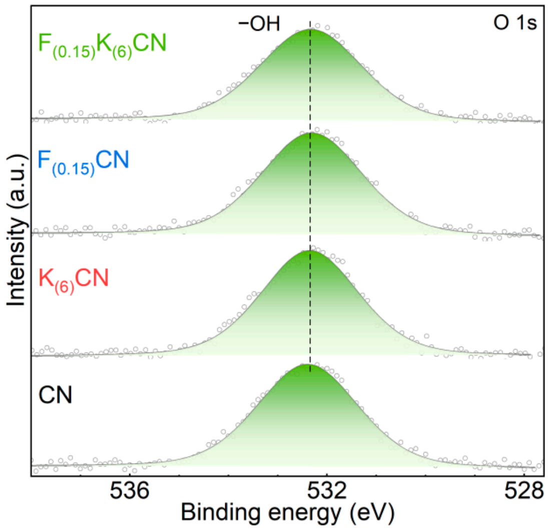

Figure S5 High resolution O 1s XPS spectra of CN, $K_{(6)}$CN, $F_{(0.15)}$CN, and $F_{(0.15)}K_{(6)}$CN.

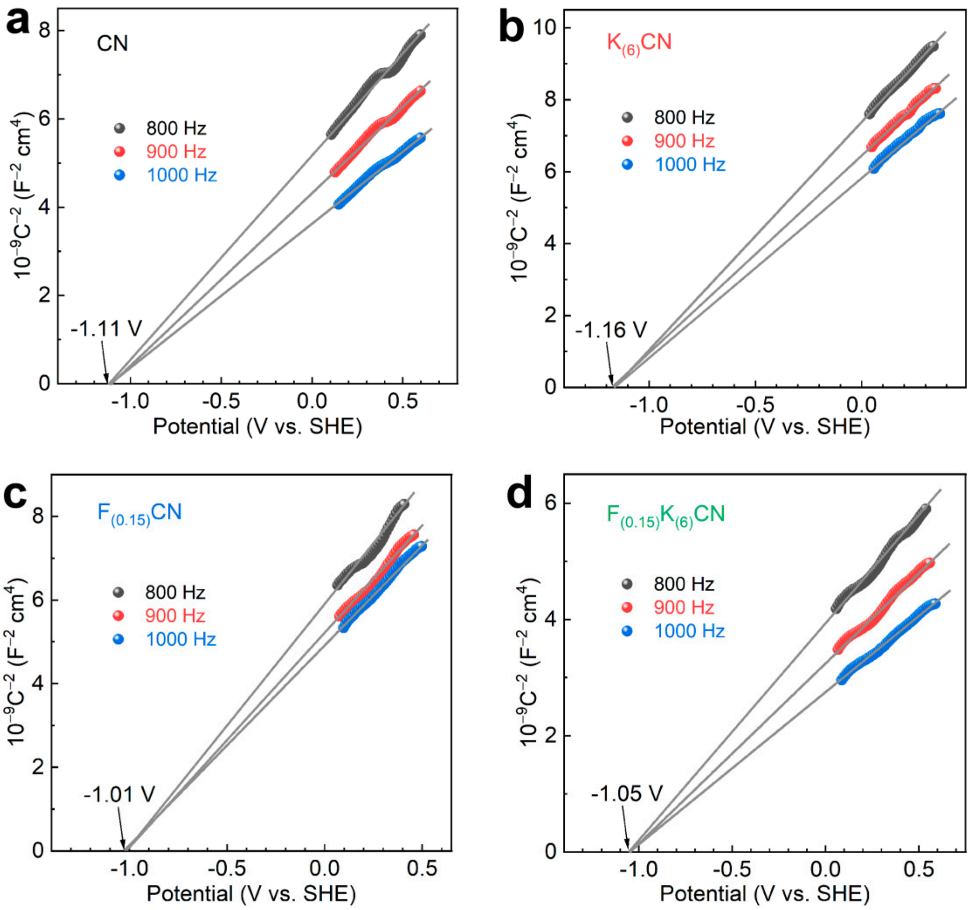

Figure S6 Mott–Schottky plots of (a) CN, (b) $K_{(6)}$CN, (c) $F_{(0.15)}$CN, and (d) $F_{(0.15)}K_{(6)}$CN.

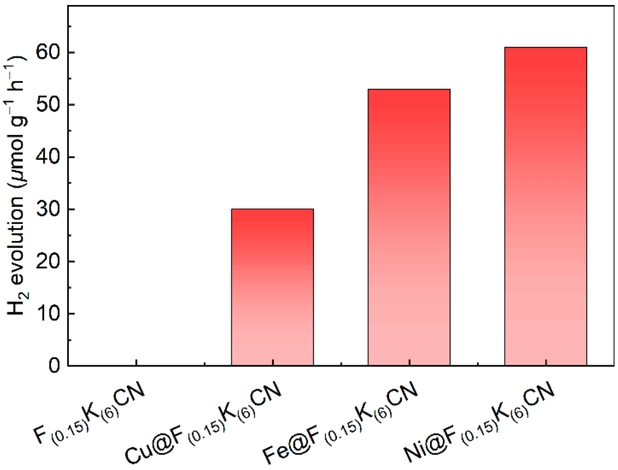

Figure S7 Photocatalytic hydrogen production activities of $F_{(0.15)}K_{(6)}$CN without co-catalyst loading, and loaded with 1 wt% of Cu, Fe, and Ni.

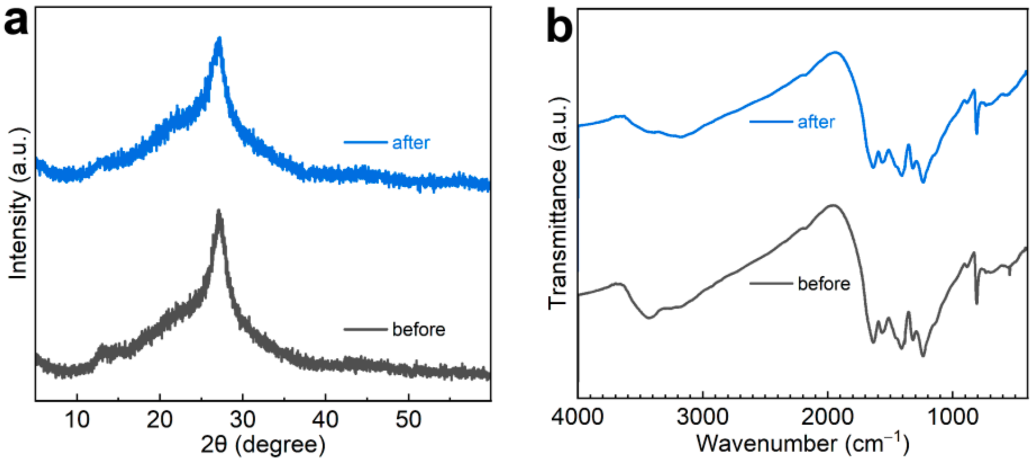

Figure S8 (a) XRD patterns and (b) FTIR spectra of $F_{(0.15)}K_{(6)}CN$ before and after the cyclic experiments.

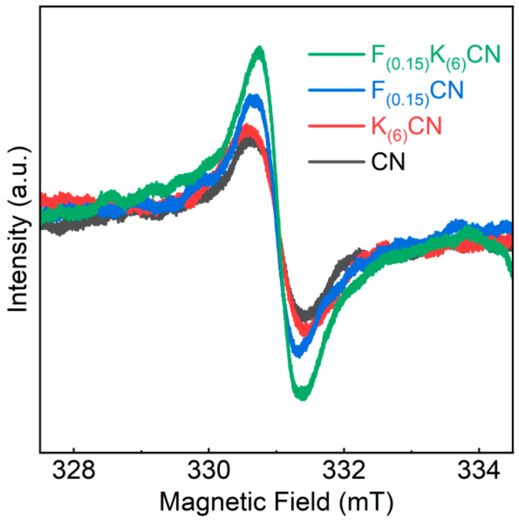

Figure S9 EPR spectra of CN, $K_{(6)}CN$, $F_{(0.15)}CN$, and $F_{(0.15)}K_{(6)}CN$.

Table S1. Photocatalytic H_2 production performance comparison between $F_{(0.15)}K_{(6)}CN$ and other reported element-doped CN photocatalysts.

Photocatalyst	Electron donor	Cocatalyst	Light source	H_2 (μmol g^{-1} h^{-1})	Ref.
$F_{(0.15)}K_{(6)}CN$	Methanol (10 vol%)	1 wt% Pt	$\lambda \geq 420$ nm	3100	**This work**
BCN-0.75	TEOA (20 vol%)	3 wt% Pt	$\lambda \geq 420$ nm	1639	[1]
IL-CN3	TEOA (10 vol%)	3 wt% Pt	full spectrum	7896	[2]
CNK	TEOA (20 vol%)	3 wt% Pt	$\lambda \geq 420$ nm	920	[3]
S@g-C_3N_4	Lactic acid (5 vol%)	1 wt% Pt	$\lambda = 420$ nm	3684	[4]
CNNSeCo	TEOA (10 vol%)	3 wt% Pt	$\lambda \geq 420$ nm	809	[5]
CN-3CoS_2	TEOA (10 vol%)	Pt	$\lambda \geq 420$ nm	195	[6]
1-C-C_3N_4	TEOA (25 vol%)	6 wt% Pt	$\lambda \geq 400$ nm	255	[7]
S-g-C_3N_4	lactic acid	2 wt% Pt	visible light	1201	[8]
B-CN/Mo_2C-35	TEOA (10 vol%)	/	$\lambda \geq 420$ nm	1696	[9]
6Sr-SCN	TEOA (17 vol%)	3 wt% Pt	$\lambda \geq 420$ nm	1399	[10]
K-CN-10	TEOA (10 vol%)	3 wt% Pt	$\lambda \geq 400$ nm	1337	[11]
K(0.05)-CN	TEOA (10 vol%)	1 wt% Pt	$\lambda \geq 420$ nm	1319	[12]
MP-CN-KI	Methanol (5 vol%)	1 wt% Pt	$\lambda \geq 420$ nm	1612	[13]
CN-0.20%Dx-25	TEOA (10 vol%)	3.84 wt% Pt	$\lambda \geq 420$ nm	1962	[14]

References

[1] X. Xia, C. Xie, B. Xu, X. Ji, G. Gao, P. Yang, Role of B-doping in g-C_3N_4 nanosheets for enhanced photocatalytic NO removal and H_2 generation, J. Ind. Eng. Chem. 105 (2022) 303−312.

[2] Z. Miao, F. Xu, B. Zhao, Y. Song, P. Sun, G. Wu, K. Xu, P. Yan, Z. Mo, H. Xu, P-doped and cyano-modified carbon nitride nanotubes for photocatalytic hydrogen evolution coupled with bisphenol A degradation, J. Colloid Interface Sci. 686 (2025) 525−534.

[3] S. Sun, J. Li, J. Cui, X. Gou, Q. Yang, Y. Jiang, S. Liang, Z. Yang, Simultaneously engineering K-doping and exfoliation into graphitic carbon nitride (g-C_3N_4) for enhanced photocatalytic hydrogen production, Int. J. Hydrogen Energy 44 (2019) 778−787.

[4] K. Ahmad, M.Q. Khan, A. Alsalme, H. Kim, Sulfur-doped graphitic-carbon nitride (S@g-C_3N_4) as bi-functional catalysts for hydrazine sensing and hydrogen production applications, Synth. Met. 288 (2022)

117100.
[5] X. Yang, Z. Tian, Y. Chen, H. Huang, J. Hu, B. Wen, In situ synthesis of 2D ultrathin cobalt doped g-C_3N_4 nanosheets enhances photocatalytic performance by accelerating charge transfer, J. Alloys Compd. 859 (2021) 157754.
[6] Y. Zhang, J. Shi, Z. Huang, X. Guan, S. Zong, C. Cheng, B. Zheng, L. Guo, Synchronous construction of CoS_2 in-situ loading and S doping for g-C_3N_4: Enhanced photocatalytic H_2-evolution activity and mechanism insight, Chem. Eng. J. 401 (2020) 126135.
[7] J. Cao, X. Jin, Z. Ma, H. Wang, Y. Xu, Y. Guo, H. Xie, J. Zhang, One-step synthesis of C quantum dots/C doped g-C_3N_4 photocatalysts for visible-light-driven H_2 production from water splitting, J. Phys. D: Appl. Phys. 55 (2022) 444008.
[8] F.A. Alharthi, I. Hasan, Improved photocatalytic hydrogen evolution using sulfur-doped graphite-like carbon nitride (S-G-C_3N_4) photocatalyst, ChemistrySelect 8 (2023) e202302369.
[9] R.-Y. Liu, L. Ding, G.-D. Yang, J.-Y. Zhang, R. Jiao, H.-Z. Sun, Hollow Mo_2C nanospheres modified B-doped g-C_3N_4 for high efficient photocatalysts, J. Phys. D: Appl. Phys. 55 (2022) 454001.
[10] F. Liu, W. Li, L. Wang, X. Rao, S. Zheng, Y. Zhang, Sulfur-and strontium-doped graphitic carbon nitride for efficient photocatalytic hydrogen evolution, ACS Appl. Energy Mater. 5 (2022) 15834−15843.
[11] Y. Wang, S. Zhao, Y. Zhang, J. Fang, Y. Zhou, S. Yuan, C. Zhang, W. Chen, One-pot synthesis of K-doped g-C_3N_4 nanosheets with enhanced photocatalytic hydrogen production under visible-light irradiation, Appl. Surf. Sci. 440 (2018) 258−265.
[12] X. Chang, H. Fan, S. Zhu, L. Lei, X. Wu, C. Feng, W. Wang, L. Ma, Engineering doping and defect in graphitic carbon nitride by one-pot method for enhanced photocatalytic hydrogen evolution, Ceram. Int. 49 (2023) 6729−6738.
[13] Y. Guo, Q. Liu, Z. Li, Z. Zhang, X. Fang, Enhanced photocatalytic hydrogen evolution performance of mesoporous graphitic carbon nitride co-doped with potassium and iodine, Appl. Catal. B Environ. 221 (2018) 362−370.
[14] J. Bi, L. Zhu, J. Wu, Y. Xu, Z. Wang, X. Zhang, Y. Han, Optimizing electronic structure and charge transport of sulfur/potassium co-doped graphitic carbon nitride with efficient photocatalytic hydrogen evolution performance, Appl. Organomet. Chem. 33 (2019) e5163.
